# Supplementary material for: Comprehensive GCMS and LC-MS/MS Metabolite Profiling of Chlorella vulgaris
Source: Mar Drugs. 2020 Jul 17;18(7):367. doi: 10.3390/md18070367 (PMC7404257; doi:10.3390/md18070367)
Supplement: Supplementary file 1 [file marinedrugs-18-00367-s001.pdf]

Supplementary materials

Neoxanthin

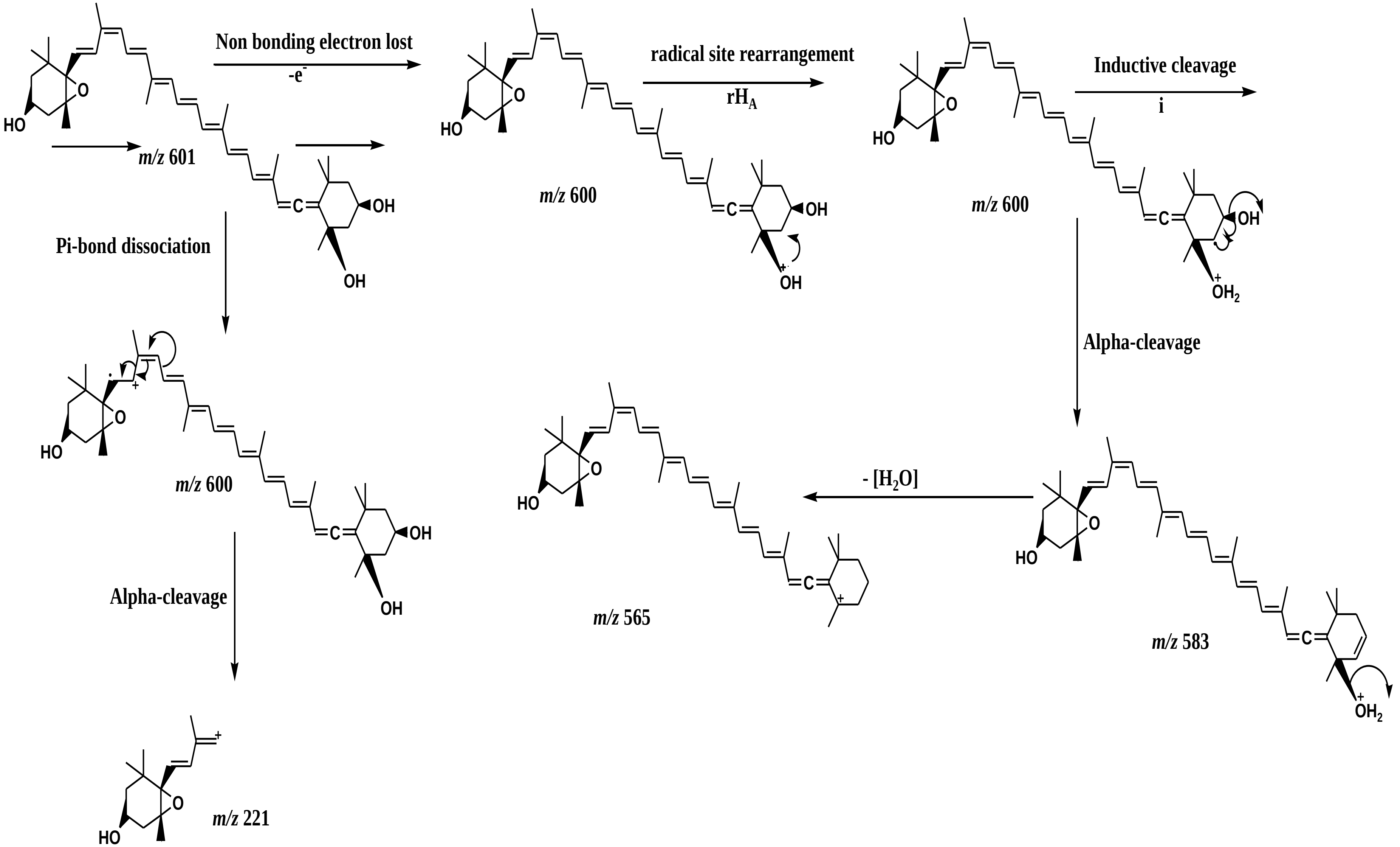

Figure S1: fragmentation pathway of neoxanthin

Violaxanthin

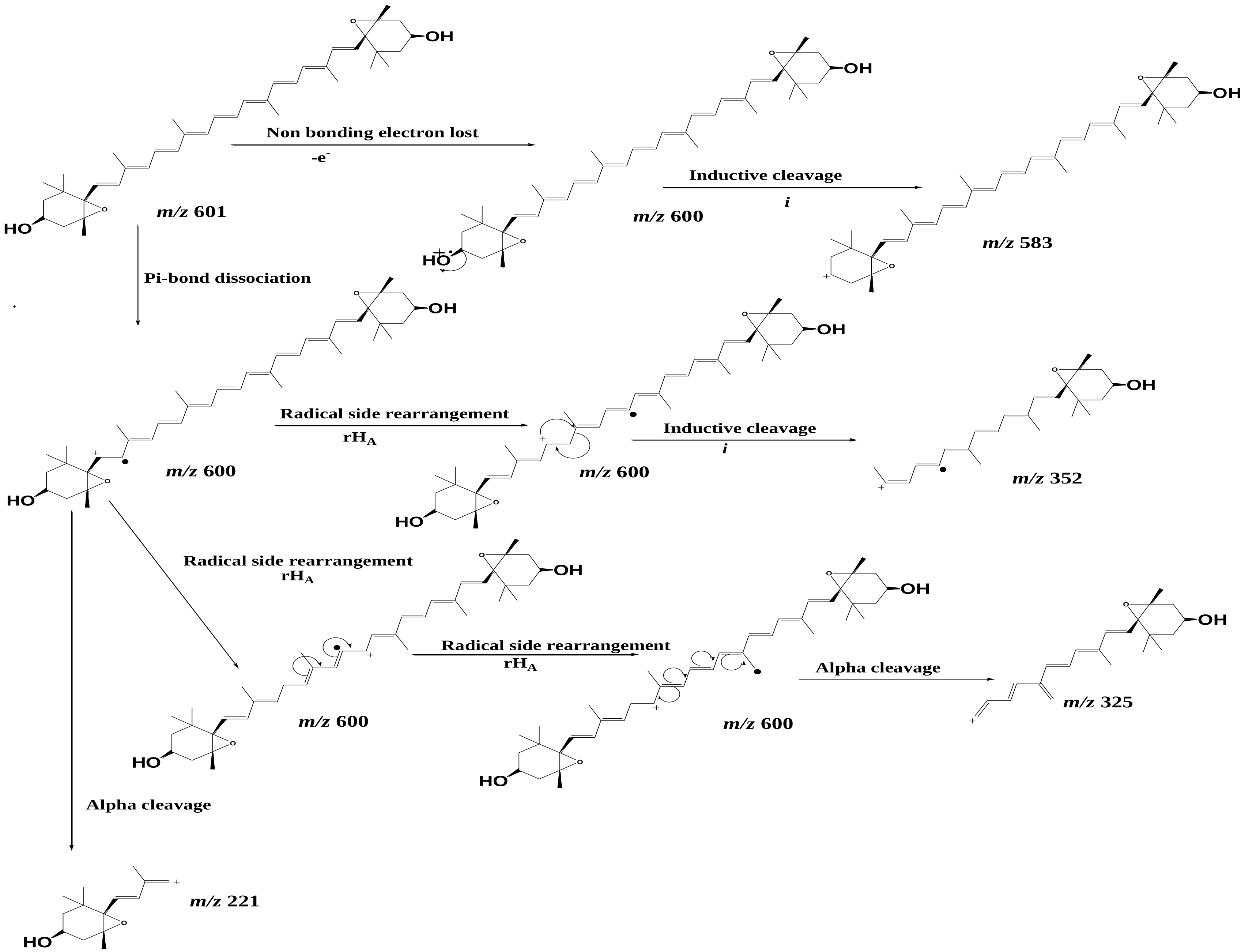

Figure S2: fragmentation pathway of violaxanthin

Lutein

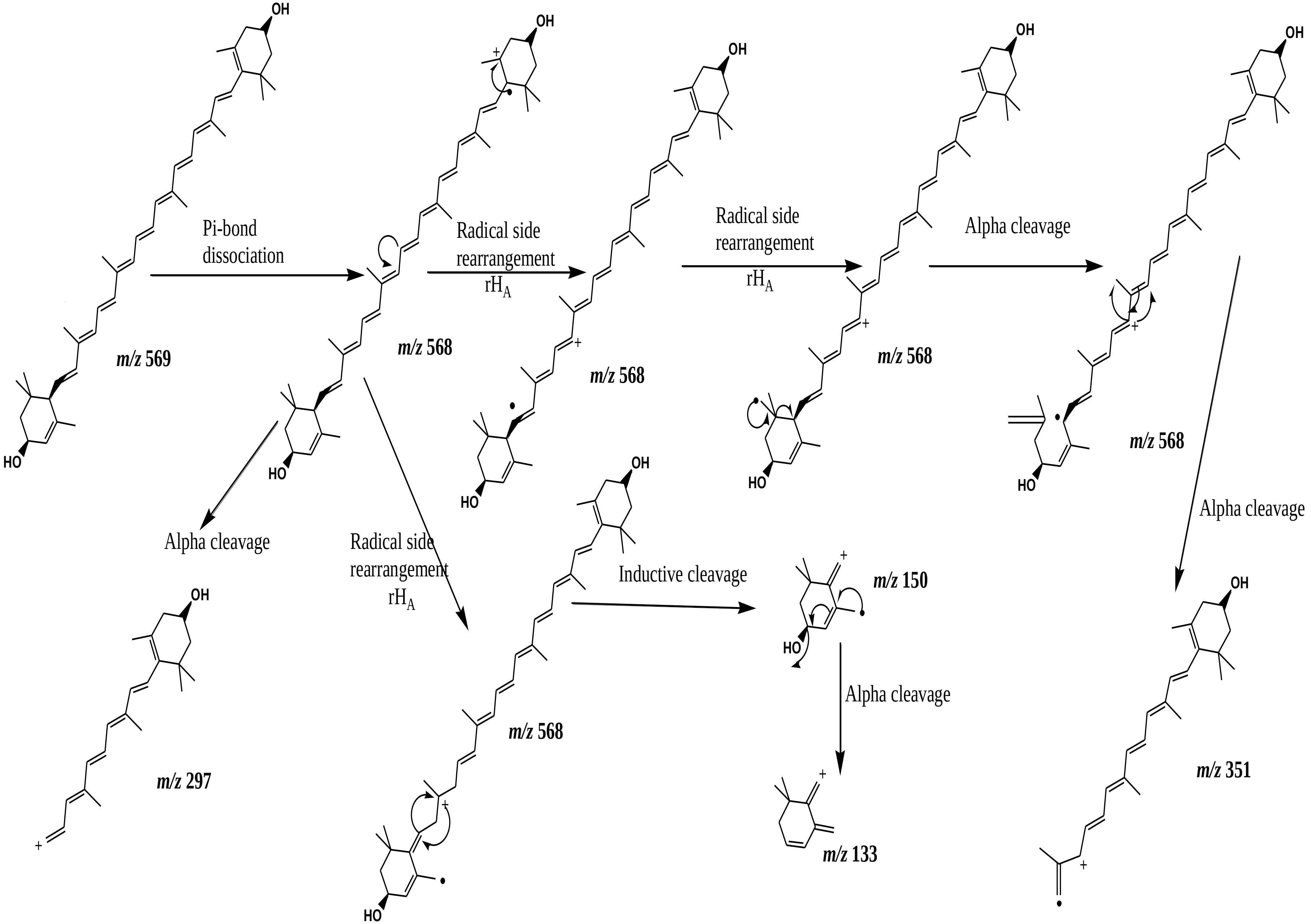

Figure S3: fragmentation pathway of lutein

β-carotene

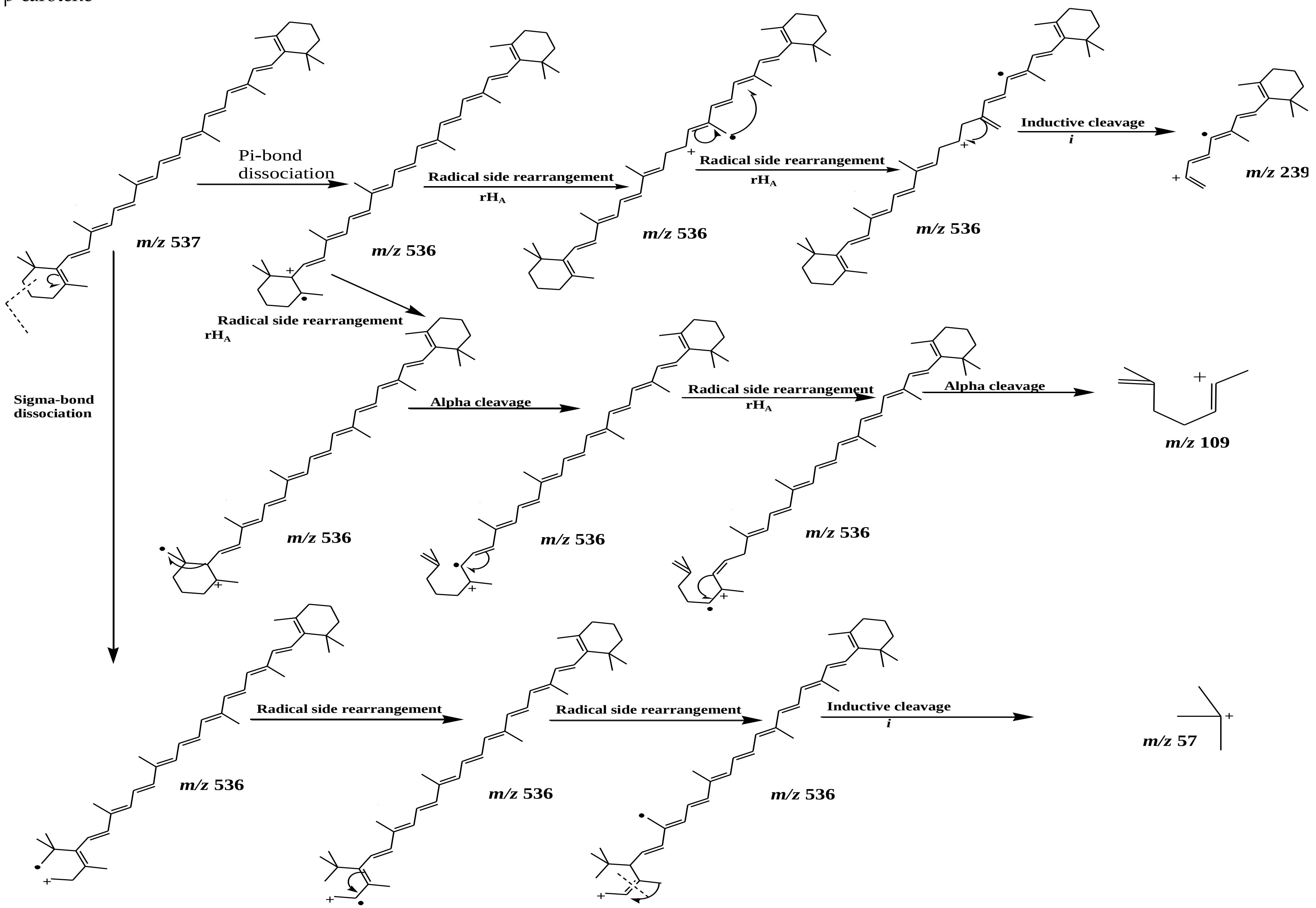

Figure S4: fragmentation pathway of β-carotene

Astaxanthin

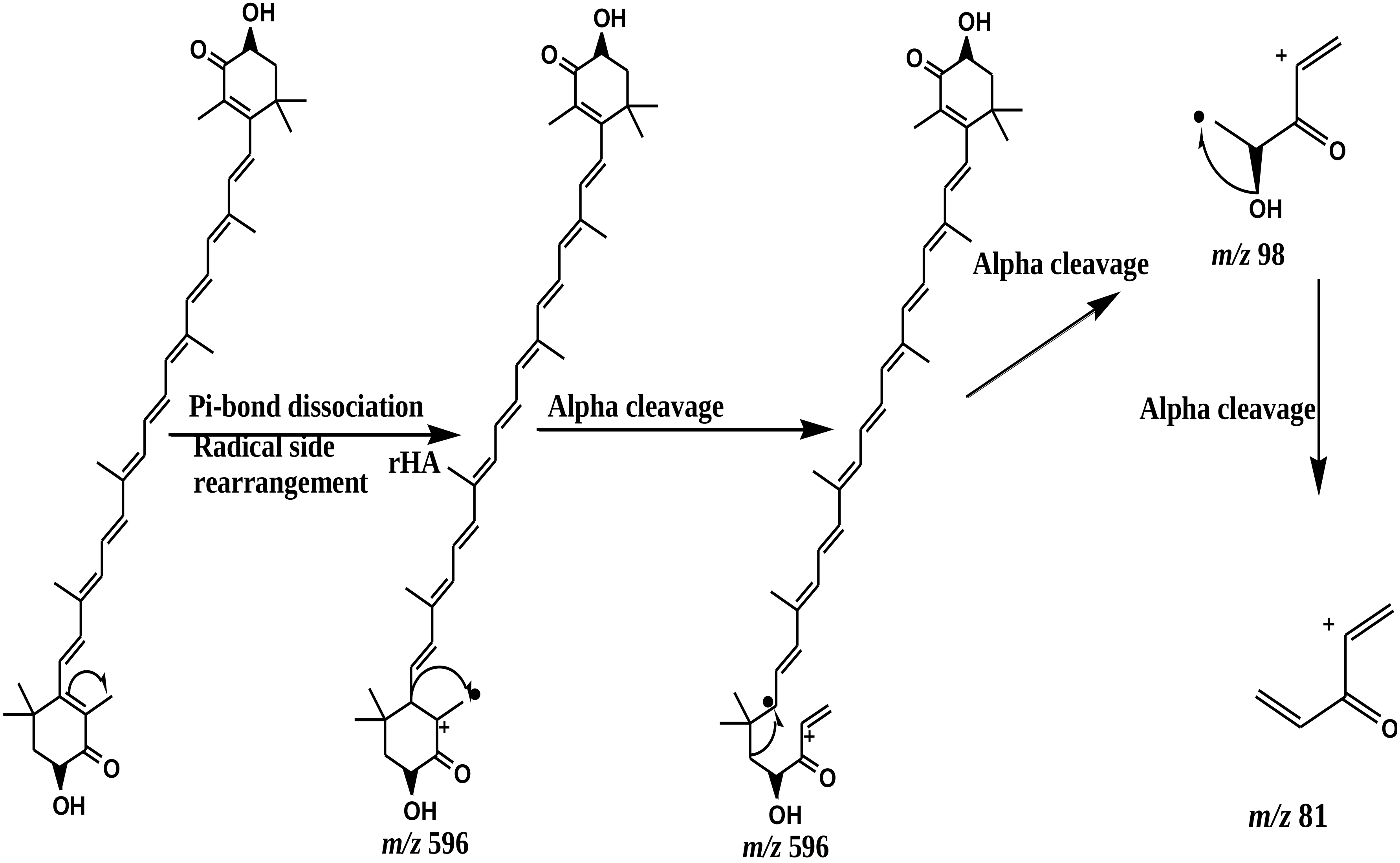

Figure S5: fragmentation pathway of astaxanthin

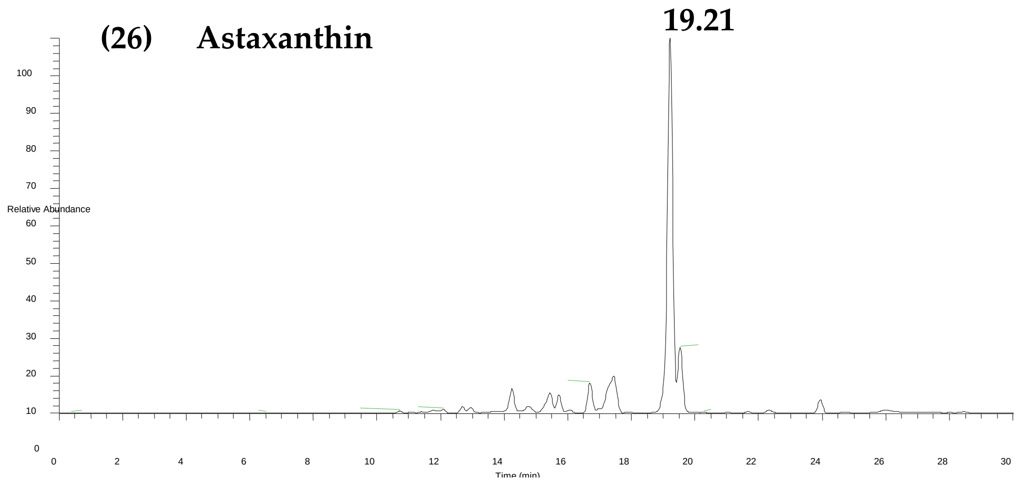

Figure S6: Extraction ion chromatogram (EIC) of (**peak 26**) Astaxanthin

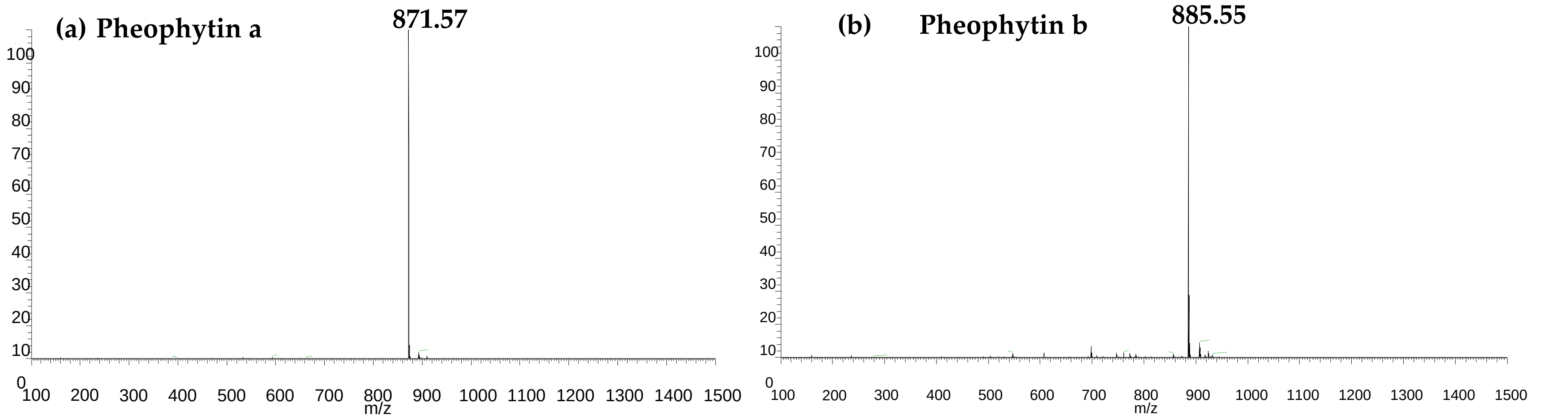

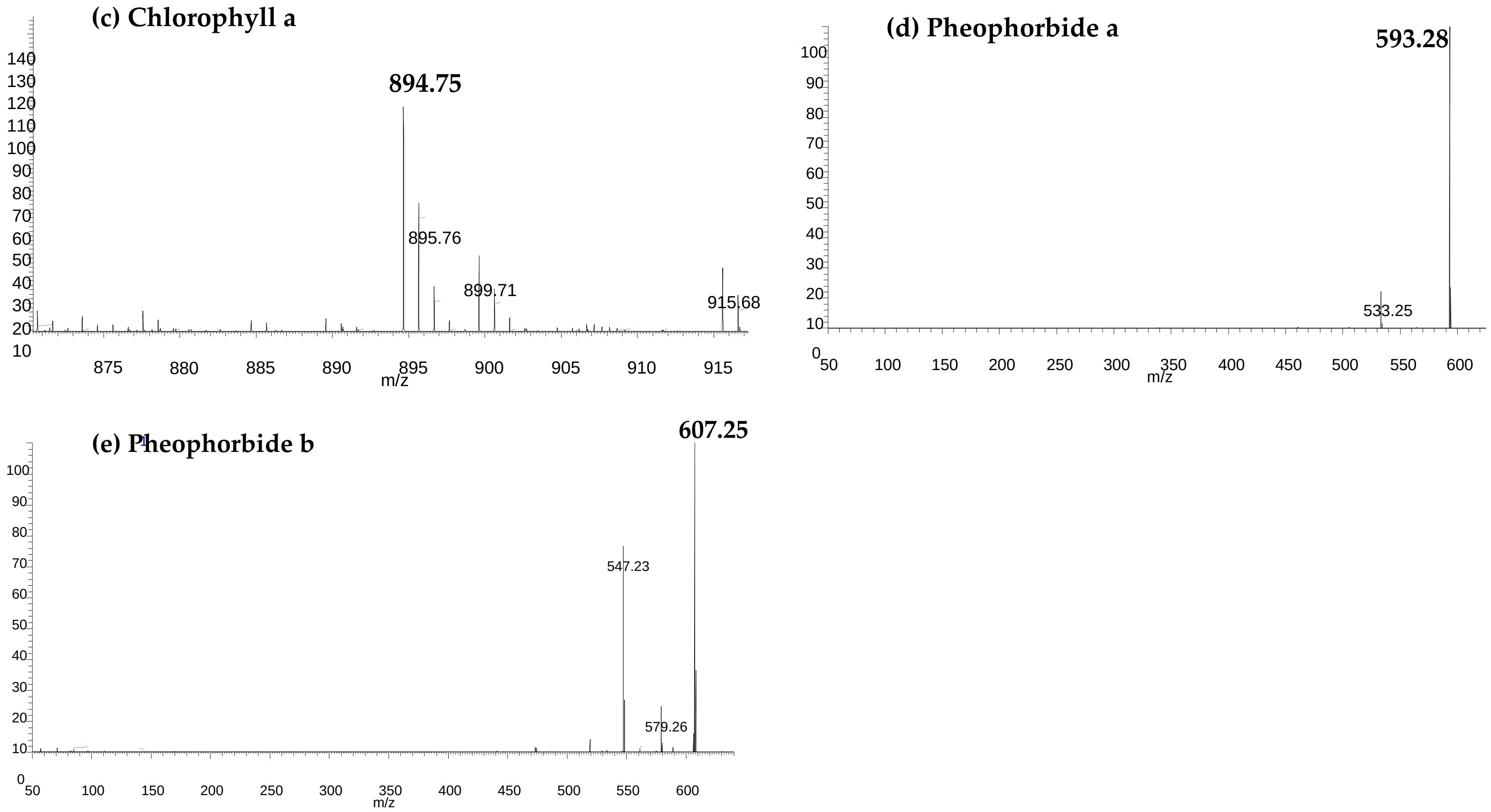

Figure S7: MS/MS of some of the representative pigments: a, pheophytin a; b, pheophytin b; c, chlorophyll a; d, pheophorbide a; e, pheophorbide b.
